# Secondary Bacterial Infections of Buruli Ulcer Lesions Before and After Chemotherapy with Streptomycin and Rifampicin

**DOI:** 10.1371/journal.pntd.0002191

**Published:** 2013-05-02

**Authors:** Dorothy Yeboah-Manu, Grace S. Kpeli, Marie-Thérèse Ruf, Kobina Asan-Ampah, Kwabena Quenin-Fosu, Evelyn Owusu-Mireku, Albert Paintsil, Isaac Lamptey, Benjamin Anku, Cynthia Kwakye-Maclean, Mercy Newman, Gerd Pluschke

**Affiliations:** 1 Noguchi Memorial Institute for Medical Research, University of Ghana, Legon, Ghana; 2 Molecular Immunology, Swiss Tropical and Public Health Institute, Basel, Switzerland; 3 University of Basel, Basel, Switzerland; 4 Reconstructive and Plastic Surgery Unit, Korle-BU Teaching Hospital, Accra, Ghana; 5 Ga South District, Ghana Health Service, Obom, Ghana; 6 Ga West District, Ghana Health Service, Amasaman, Ghana; 7 Department of Microbiology, University of Ghana Medical School, University of Ghana, Korle-Bu, Ghana; University of Tennessee, United States of America

## Abstract

Buruli ulcer (BU), caused by *Mycobacterium ulcerans* is a chronic necrotizing skin disease. It usually starts with a subcutaneous nodule or plaque containing large clusters of extracellular acid-fast bacilli. Surrounding tissue is destroyed by the cytotoxic macrolide toxin mycolactone produced by microcolonies of *M. ulcerans*. Skin covering the destroyed subcutaneous fat and soft tissue may eventually break down leading to the formation of large ulcers that progress, if untreated, over months and years. Here we have analyzed the bacterial flora of BU lesions of three different groups of patients before, during and after daily treatment with streptomycin and rifampicin for eight weeks (SR8) and determined drug resistance of the bacteria isolated from the lesions. Before SR8 treatment, more than 60% of the examined BU lesions were infected with other bacteria, with *Staphylococcus aureus* and *Pseudomonas aeruginosa* being the most prominent ones. During treatment, 65% of all lesions were still infected, mainly with *P. aeruginosa*. After completion of SR8 treatment, still more than 75% of lesions clinically suspected to be infected were microbiologically confirmed as infected, mainly with *P. aeruginosa* or *Proteus miriabilis*. Drug susceptibility tests revealed especially for *S. aureus* a high frequency of resistance to the first line drugs used in Ghana. Our results show that secondary infection of BU lesions is common. This could lead to delayed healing and should therefore be further investigated.

## Author Summary

Buruli ulcer (BU) can lead to large ulcerative lesions due to extensive skin loss caused by the necrotizing effect of the main virulence factor mycolactone. For a long time the general perception was that BU lesions are not infected by other bacteria because of a postulated antimicrobial effect of the macrolide toxin, mycolactone. In this study, we analyzed laboratory confirmed BU lesions before, during, and after streptomycin/rifampicin treatment. Contrary to popular belief, our findings show that BU lesions are frequently co-colonized with other potential bacterial pathogens before, during, and after antibiotic treatment. For example, 75% of cases that were clinically indicative of being infected after treatment were microbiologically confirmed as infected. Most microbiologically infected cases were also confirmed by histopathological analysis. The most prominent bacterial species isolated included *Pseudomonas aeruginosa*, *S. aureus*, and *P. mirabilis*. When we tested the isolates against first line drugs used in Ghana, the isolates were found to be resistant to most of these drugs. This study indicates that wound care practices need to be improved and that wound infection may be a common cause of wound healing delay in BU.

## Introduction

Buruli ulcer (BU) caused by *Mycobacterium ulcerans* is a necrotizing skin disease that affects mainly impoverished communities in Western and Central Africa. It is the third most common mycobacterial disease of humans after tuberculosis and leprosy. BU lesions are characterized by extensive necrosis and minimal pain and inflammation [1], [2]. The pathogenesis of the disease is believed to be initiated by the inoculation of *M. ulcerans* into the subcutaneous layer of the skin, which may be facilitated by trauma or an insect vector. Most BU lesions are found at the extremities and contain extracellular clusters of acid-fast bacilli (AFB) in the subcutaneous fat tissue. The incubation period seems to be highly variable, and has been estimated to range from two weeks to three years, with an average of two to three months [3]. The disease begins typically as a painless nodule under the skin and gradually enlarges and erodes through the skin surface, leaving a well-demarcated ulcer with a necrotic slough in the base and widely undermined edges [3], [4].

Traditionally, the mainstay treatment of BU was surgical removal of infected tissues followed by skin grafting [1]. This led to long hospital stays with the accompanied social problems of losses of school time by children and a large economical burden directly and indirectly to the affected families. Since 2006, after a pilot study in Ghana, the first line treatment of BU is SR8 (eight weeks of streptomycin daily injections and oral therapy with rifampicin) [5]–[7]. This has reduced surgery to an adjunct procedure in BU management. The general perception is that this treatment modality will reduce the length of stay in health facilities, since it removes the fear of surgery and encourages early reporting to the formal health sector for treatment. SR8 makes a decentralization of treatment possible, since staff of peripheral health facilities can administer streptomycin injections.

The pathogenesis of BU is mediated mainly by a polyketide derived macrolide toxin, named mycolactone, with potent tissue necrotizing [8] and immunosuppressive activities [9], [10]. Mycolactone produced by clusters of *M. ulcerans* leads to the destruction of the surrounding soft skin tissue and to the formation of devitalized, avascular tissue and ‘necrotic slough’ at the wound bed, which is very characteristic of BU [11]. The necrotic tissue could provide an ideal medium for bacterial growth and may disturb and delay wound healing. While there is a popular belief that secondary infections of BU lesions are rare, because mycolactone has antimicrobial activities, there is no published evidence base for this.

It is controversial, whether bacteria present in wounds contribute to delays in wound healing, because wounds generally harbor transient microorganisms (contamination) [12]. The surfaces of wounds have microbial populations at each stage of healing and some of the bacteria may be involved in mutually beneficial relationships with the host preventing more virulent organism from infecting deeper tissues. Such beneficial organisms include coagulase negative *Staphylococcus* and *Corynebaceria* species [12]–[14]. These contaminating organisms are derived from the normal flora of the surrounding skin, mucous membranes or from external environmental sources. Usually the immune defense mechanisms of the host can contain these contaminants with no harm and negative consequence to wound healing. However, some of the contaminating organisms can also go on to colonize, massively multiply and delay wound healing. Only when a critical concentration of these microorganisms is reached, signs of infection including erythema, pain, increase in temperature, odor and discoloration of granulation tissue are observed. Therefore assessment of wound infection has to be based both on the density of microorganisms as well as on the presence of specific pathogenic species [15], [16]. *Staphylococcus aureus*, *Pseudomonas aeruginosa*, and beta-hemolytic streptococci are regarded as primary indicators for a delayed healing and infection in both acute and chronic wounds. Bacterial loads exceeding 10^6^ colony forming units (CFU)/g of tissue or tissue fluid, accumulations of pus cells and presence of specific pathogenic organism are being used as indicators for wound infection in contrast to wound contamination [16]–[19]. Factors predisposing a wound to infection include the non-observance of principles of good hygienic procedures during dressing and the presence of necrotic tissue or slough within the wound margin [13], which is commonly found in BU lesions. The extent of secondary infections in BU and their contribution to frequently observed delays in healing has not been studied so far. Here we have analyzed BU lesions before, during and after antimicrobial treatment for the presence of secondary infection.

## Materials and Methods

### Study participants and sample collection

The participants involved in the study were recruited from the Amasaman District Hospital and the Obom Health Centre in the Ga-West and Ga South Municipality, respectively. The participants were all laboratory confirmed BU cases and the analyzed samples fall into three main categories: 1) samples from 53 BU patients recruited consecutively before treatment; 2) samples from 20 BU patients recruited consecutively between four and six weeks after start of SR8 and 3) samples from 31 BU patients whose lesions were clinically suspected of secondary infection after SR8 treatment. Some of the participants overlapped in some of the categories: 71 of the participants were sampled once for analysis, 12 twice and 3 thrice within the study period, thus in total 104 individual samples, 84 swabs and 20 tissue samples, from 86 participants were analyzed. The swabs were obtained from 52 cases before treatment, 20 cases during and 12 cases after treatment and analyzed microbiologically (Table S1). The tissue samples for histopathological analysis were obtained from one case before treatment and 20 cases after treatment. Except for one sample taken after treatment, all tissue samples were also analyzed microbiologically (Table S1).

A detailed questionnaire was used to obtain standard demographic data, document the clinical presentation of lesions and other lesion characteristics. Altogether the study involved 86 participants comprising 32 (37%) females and 54 (63%) males. The females' age ranged between two and 72 years and the males were between four months and 82 years. Median age for both groups was 33 years. Seventy-seven of the cases had lesions located on the limbs, three in the head and neck region, and one each located on the buttocks, armpit and back respectively; the lesion location of three participants was not documented.

**Figure 1:**
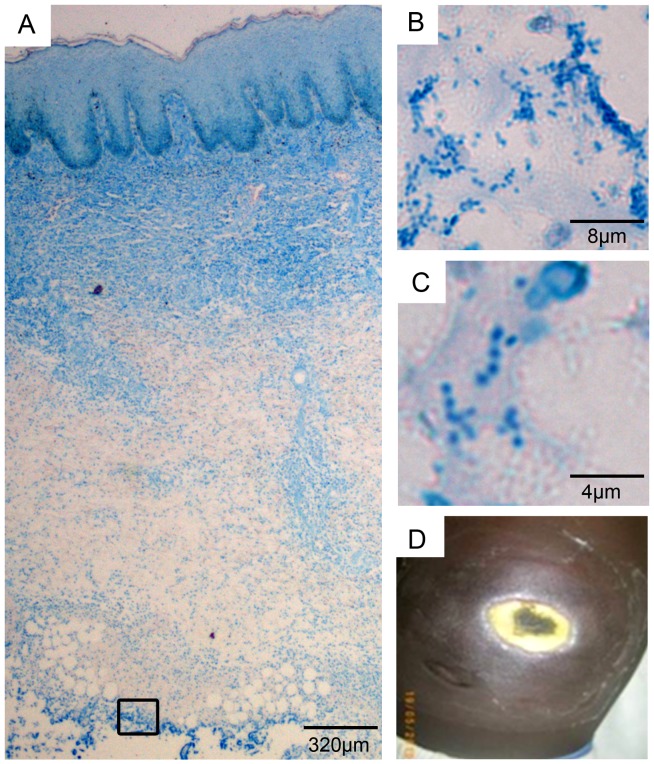
Histopathological analysis of tissue excised before start of SR8 treatment. Histological sections were stained with Ziehl-Neelsen (acid fast bacteria) and methylene blue (DNA, secondary infection). A: Overview over excised tissue specimen revealing infection at the lower end of the specimen (box), as well as BU characteristic histopathological features, including fat cell ghosts, necrosis and epidermal hyperplasia. B/C: higher magnification revealing the presence of cocci. D: clinical presentation of the lesion on the belly.

Only 2/86 patients were pre-ulcerative. These lesions, one nodule and one plaque, were sampled later during surgery. The remaining 84 patients had ulcers; 78 of them had only ulcers, one had an ulcer and a nodule, three had ulcers with edema, and two had ulcers with osteomyelitis. Based on the judgment of the responsible clinician, surgical debridement was performed for 1 patient prior to treatment and for 20 patients after completion of SR8. Biopsy samples were collected in each instance for histopathological analysis (Figure 1).

Laboratory confirmation of BU disease was done by IS*2404* PCR and Ziehl-Neelsen microscopy as previously described [20], [21]. Three swab samples were collected from clinically suspected ulcerative cases before treatment; one for *IS2404*-PCR based confirmation of BU, one for preparation of a direct smear for microscopic examination for the detection of bacteria and neutrophils after Gram staining (Figure S1), and the third was inserted into a sterile tube containing 3 ml of PBS for enumeration of the bacterial burden and the isolation of specific bacterial species. All swab specimens were collected from the undermined edges of lesions by first moistening the swab with sterile PBS using the Levine method of collecting swab specimen [22]. This has been found to be the best method for taking swabs as it is more reflective of tissue bio-burden as compared to other methods [23]. After cleaning the wound surface with normal saline, a swab was rotated over a 1 cm^2^ area with sufficient pressure to collect the fluid from within the wound tissue.

From cases that were sampled during treatment and those that were clinically suspected of having a bacterial infection after completion of SR8, three swab specimens were collected before surgery, and treated as above, except for the procedures for the laboratory confirmation of BU disease by PCR, since all cases had been previously confirmed as BU within the framework of a bigger study. From SR8 treated patients that underwent surgical management, tissue sample were analyzed if there was clinical suspicion of a secondary bacterial infection. While one sample was aseptically transferred into a clean sterile tube for enumeration of the bacterial load and species identification, a second sample was directly transferred into 10% neutral buffered formalin for histopathological analysis.

The samples for bacteriological analysis were placed in an ice chest with ice packs to prevent bacterial multiplication and transported to the Bacteriology Department of the Noguchi Memorial Institute for Medical Research (NMIMR) for analysis, Tissue samples for were shipped to the Swiss Tropical and Public Health Institute for histopathological analysis.

### Ethics statement

Ethical clearance was obtained from the institutional review board of the Noguchi Memorial Institute for Medical Research (Federal-wide Assurance number FWA00001824). The procedures for sampling in this study were essentially the same as those used in routine management of BU in Ghana. However, written informed consent was collected from all participants before study inclusion. In the case of children below sixteen years, written informed consent was collected from their parents or guardians. Patients were assured of the confidentiality of all information collected during the study.

### Enumeration of the bacterial load and isolation of bacteria

When swab samples reached the microbiology laboratory, the volume of PBS was topped up to 5 ml and both the swab and the PBS were transferred into a sterile glass tissue culture tube containing glass beads. The tubes were vortexed for about two minutes to dislodge any particles that were sticking to the swabs. Using the resulting stock suspension, serial dilutions from 10^−2^ to 10^−6^ were prepared.

Hundred microlitres of serial dilutions of the swab or tissue suspensions were transferred into sterile Petri dishes and inoculated by the pour plate method using Plate Count Agar for total aerobic counts. The agar was left on the lab bench to set after which it was incubated at 37°C for 18–24 hours. The remaining 10^−1^ dilution of the suspension was centrifuged at 8,000 g for 25 minutes and after decanting, the pellet was inoculated onto MacConkey, Blood and Chocolate agar and incubated under aerobic conditions. The aerobic agar plates were examined after 24 hours and growing colonies were subcultured on Blood and MacConkey agar plates to obtain pure cultures.

After incubation, the plates were examined using a colony counting chamber (Gallenkamp, UK) and those with colony counts between 30 and 300 were selected for computing CFU/ml or CFU/g, respectively, by multiplying the counts by the dilution factors. The lesion from which the sample was taken was classified as clean, contaminated or infected as indicated in the data analysis section.

For tissue specimen, one gram of sample was weighed in a sterile plastic stomacher bag. Nine milliliters of PBS were added, samples were macerated in a stomacher and the resulting suspension was transferred into a sterile test-tube. Using this stock suspension, serial dilutions were prepared and plated out.

### Species identification of bacterial isolates

Distinct bacterial colonies from the Blood and MacConkey agar plates were purified on Nutrient agar plates for identification. Bacterial isolates were Gram stained [24] and identified by biochemical tests as well as by molecular methods. Gram negative rod isolates were characterized by cytochrome oxidase analysis, and with Analytical Profile Index (API 20E) strips (bio-M**é**rieux SA, Marcy-l'E'toile, France) according to the manufacturer's instructions. Gram positive cocci were analyzed after Gram staining using the catalase test to differentiate between *Staphylococcus spp.* and *Streptococcus spp.* In order to further discriminate the catalase positive Gram positive cocci and especially to identify *Staphylococcus spp.*, the Staphylase kit Prolex Latex Agglutination System (Pro-Lab Diagnostics) was used. Gram positive bacteria were further characterized using the Hain Lifescience Genotype Product series for Gram positive bacteria Genotype BC Gram positive version 3.0 and Genotype staphylococcus version 2 test kits (Hain Lifescience, Germany). Where species identification failed with the analytical profile index and the other biochemical assays, identification was achieved by MALDI-TOF mass spectrometry [25].

### Drug susceptibility testing

Susceptibility of isolates to specific drugs was tested using the Kirby-Bauer disc diffusion method on Mueller Hinton agar [26]. Sensitivity was tested against antibiotics such as Cotrimoxazole, Ampicillin, Tetracycline, Ciprofloxacin, Amikacin, Gentamicin, Penicillin, Erythromycin, Cefuroxime, Cefixime, Ceftriaxone, Chloramphenicol and Flucloxacillin. In addition Gram positive cocci were tested against methicillin and vancomycin. The results of isolation and drug sensitivity tests were provided to the treating clinician at the collaborating health facility. Since the locally available disc systems varied in coverage, some antibiotics were only tested with a subset of isolates. One limitation of this study is that we did not test for susceptibility against streptomycin and rifampicin.

### Histopathology

Histopathological analysis was done for all SR8 treated patients needing surgical management and presenting with a lesion clinically suspicious for secondary infection. Surgically excised tissue samples were immediately fixed after excision in 10% neutral-buffered formalin for 24 h at room temperature to maintain tissue structures. Afterwards samples were directly transferred to 70% ethanol for storage and transport. Tissue specimens were subsequently dehydrated, embedded into paraffin, and cut into 5 µm sections. After deparaffinization and rehydration, sections were stained with Ziehl-Neelsen/Methyleneblue (ZN) according to WHO standard protocols [3]. In this staining AFB appear pink and other bacteria are stained blue. Tissue sections were analyzed with a Leica DM2500 Microscope and pictures were either taken with a Leica DFC 420C camera or with an Aperio ScanScope XT.

### Analysis of recycled bandages

Recycled bandages from fifteen confirmed BU cases were collected conveniently before wound dressing for microbiological analysis. Ten grams bandage was weighed, added to 90 ml of sterile PBS and macerated with a laboratory blender to give a 10^−1^ dilution. Using this suspension, serial dilutions from 10^−2^ to 10^−6^ were prepared. Hundred microlitres of these serially diluted suspensions were transferred into sterile Petri dishes and inoculated by the pour plate method using Plate Count Agar for total aerobic counts. Bacterial enumerations were performed as described above. In addition the left over suspension was centrifuged at 3,000 g for 20 mins and the resulting pellet was plated for bacterial isolation.

### Data analysis

The values obtained from plate counts were computed into CFU/ml for wound exudates (swabs) or CFU/g for tissue sample. The antibiogram of each isolate was interpreted according to the manufacturer's specification as resistant, intermediate or susceptible. The percentages of cases in each category were then computed.

### Classification of wounds

Lesions were classified microbiologically as clean if no bacteria were isolated, as contaminated if bacterial counts were <10^6^ CFU/g or ml and as infected if counts were >10^6^ CFU/g or ml of specimen.

Lesions were clinically classified as infected based on the following criteria: 1. friable, bleeding granulation tissue despite appropriate care and management; 2. purulent discharge (yellow or green) from wound or drain placed in wound; 3. pain or tenderness, localized swelling (edema), or redness/heat; 4. tissue necrosis; 5. skin grafting failure; abnormal odor coming from the wound site; delayed healing not previously anticipated. Twenty-four of the patients clinically classified as infected were in-patients and seven were out-patients, who were reporting twice a week for wound dressing. During wound dressing, the wounds were cleaned with normal saline to wash away debris. Wounds that appeared necrotic or had an offensive odor were cleaned again with vinegar and dressed with povidine iodine.

## Results

### Bacterial infection of lesions from PCR-confirmed BU patients before and during SR8 treatment

Swab samples of 52 consecutively recruited IS*2404* PCR confirmed BU cases with ulcerative lesions were sampled before the commencement of SR8 treatment. Samples from three participants (5.7%) did not yield any aerobic growth on plate count agar (Table 1). Seventeen (32.1%) of the lesions with total CFU counts of 1.7×10^3^ to 9.0×10^5^ CFU/ml (average 3.2×10^5^ CFU/ml) were microbiologically classified as contaminated. Microbiologically Infected lesions were observed in 33/52 patients (63.5%); aerobic counts from this group ranged between 1.0×10^6^ to 3.5×10^9^ CFU/ml with an average value of 1.1×10^9^ CFU/ml. The most frequently identified bacterial species from the infected lesions prior to start of treatment (Table 1) were *S. aureus* (n = 9; 21.4%), *P. aeruginosa* (n = 7; 16.7%) and *P. mirabilis* (n = 6; 14.3%).

**Table 1:** Spectrum of bacterial species isolated from BU lesions before, during or after SR8 treatment.

Time of sampling	Clean wounds	Contaminated wounds	Infected wounds	Spectrum of bacteria isolates from infected cases n (%)
Before SR8 Treatment (n = 53)	3 (6%)	17 (32%)	33 (62%)	9 (22%) *Staphylococcus aureus*
				7 (17%) *Pseudomonas aeruginosa*
				6 (15%) *Proteus mirabilis*
				3 (7%) Coagulase negative Staph.
				3 (7%) *Chryseomonas luteola*
				2 (5%) *Enterobacter cloacae*
				2 (5%) *Klebsiella pneumonia*
				2 (5%) *Escherichia coli*
				1 (2%) *Streptococcus dysgalactia*
				1 (2%) *Providencia stuartii*
				1 (2%) *Staphylococcus haemolyticus*
				1 (2%) *Morganella morganii*
				1 (2%) *Streptococcus agalactia*
				1 (2%) *Staphylococcus warneri*
				1 (2%) *Proteus vulgaris*
During SR8 Treatment (n = 20)	0 (0%)	7 (35%)	13 (65%)	6 (38%) *Pseudomonas aeruginosa*
				2 (13%) *Proteus mirabilis*
				1 (6%) *Staphylococcus warneri*
				1 (6%) Coagulase negative Staph.
				1 (6%) *Enterobacter cloacae*
				1 (6%) *Providencia stuartii*
				1 (6%) *Staphylococcus haemolyticus*
				1 (6%) *Enterococcus gallinum*
				1 (6%) *Flavibacterium oryzihabitans*
				1 (6%) *Chryseomonas luteola*
After SR8 Treatment (n = 31; clinically diagnosed for secondary infection)	0 (0%)	7 (23%)	24 (77%)	8 (32%) *Pseudomonas aeruginosa*
				5 (20%) *Proteus mirabilis*
				3 (12%) *Staphylococcus aureus*
				2 (8%) *Escherichia coli*
				2 (8%) *Providencia stuartii*
				2 (8%) *Klebsiella pneumoniae*
				1 (4%) Coagulase negative Staph.
				1 (4%) *Alcaligenes faecalis*
				1 (4%) *Acinetobacter sp*

The responsible clinician decided to perform wound debridement of one of the lesions prior to SR8 initiation, since it showed clinical signs of a strong secondary infection (Figure 1D). A biopsy specimen was taken and the histopathological analysis of the tissue sample (Figure 1A–C) revealed, typical hallmarks of BU, such as fat cell ghosts, tissue necrosis and epidermal hyperplasia (Figure 1A). In addition, clusters of cocci were observed in the subcutaneous tissue between the fat cells (Figure 1A box, B, C). This area probably represents the tissue base of the undermined edges. These findings correlated well with the microbiological analysis, since *S. aureus* was isolated in large numbers from the lesion (1.2×10^9^ CFU/g).

Twenty laboratory-confirmed BU cases were consecutively sampled between four and six weeks after start of SR8 treatment and analyzed for infection of the lesions. Of these lesions, 7/20 (35.0%) and 13/20 (65.0%) were microbiologically classified as contaminated or infected, respectively; clean wounds were not observed (Table 1). The aerobic bacterial load ranged between 1.5×10^6^ and 3.5×10^9^ CFU/ml, with an average value of 5.6×10^8^ CFU/ml for the microbiologically infected lesions. The contaminated lesions had counts between 5.2×10^3^ and 7.3×10^5^ CFU/ml (average 3.3×10^5^ CFU/ml). Also here *P. aeruginosa* (n = 6; 35.3%) and *P. mirabilis* (n = 2; 11.8%), but not *S. aureus* (n = 0), were the most frequently identified bacterial species isolated from the infected lesions (Table 1).

### Bacterial infection of BU lesions with clinical signs of infection after completion of SR8 treatment

Thirty-one BU lesions with clinical signs of secondary bacterial infection after completion of SR8 treatment were sampled for laboratory investigation. Clinical signs indicative for secondary infection were documented for 28 of them and included: localized pain (28/28), viscous/purulent discharge (28/28), edema (5/28) and localized heat (4/28). In addition, delayed healing not previously anticipated (17/28), offensive odor (15/28) and discoloration of tissues both within and at the wound margins (3/28) were regarded as signs of secondary infection (Table 2). The time at which infection was detected ranged from a few weeks to fifteen months after completion of SR8.

**Table 2:** Presentation of wounds that were clinically infected after SR8 compared to microbiology and histology findings.

BU Case	Clinical Presentation									Microbiological category	Species	Histopathology	Location (in the tissue)
	Odor	Pain	Green discharge	Yellow discharge	Necrotic tissue	Bloody discharge	WHD^1^	Edema	SGF^2^				
**01**	Yes	Yes	No	Yes	Yes	No	Yes	No	Yes	Infected	Mixed growth	Rods/Cocci	Stratum corneum
**02**	Yes	Yes	No	Yes	Yes	No	Yes	No	Yes	Infected	*Providencia stuartii*	None	
**03**	No	Yes	No	Yes	Yes	No	Yes	Yes	No	Infected	*Klebsiella pneumoniae*	Rods	Stratum corneum
**04**	No	Yes	No	Yes	No	No	No	No	No	Infected	Mixed growth/*S. aureus*	Rods/Cocci	Stratum corneum
**05**	No	Yes	No	Yes	Yes	No	No	No	No	Infected	*Pseudomonas aeruginosa*	n/d	
**06**	Yes	Yes	No	Yes	Yes	No	Yes	No	No	Infected	*Klebsiella pnuemoniae, Coagulase negative Staphylococcus species*	n/d	
**07**	Yes	Yes	No	Yes	Yes	Yes	Yes	Yes	No	Infected	*E. coli, Pseudomonas aeruginosa, S. aureus*	n/d	
**08**	No	Yes	No	Yes	Yes	No	No	No	No	Contaminated	*Pseudomonas aeruginosa*	None	
**09**	Yes	Yes	Yes	Yes	Yes	No	Yes	No	No	Infected	*Pseudomonas aeruginosa*	Rods	Ulcer surface
**10**	Yes	Yes	No	Yes	Yes	No	No	No	No	Infected	Proteus mirabilis	None	
**11**	Yes	Yes	No	Yes	Yes	No	Yes	Yes	No	Infected	*Proteus mirabilis, Enterobacter cloaccae*	n/d	
**12**	Yes	Yes	No	Yes	Yes	No	Yes	No	No	Infected	*Proteus mirabilis*, mixed growth	None	
**13**	No	Yes	No	Yes	Yes	No	Yes	No	No	Infected	*Proteus mirabilis*	n/d	
**14**	Yes	Yes	No	Yes	Yes	Yes	Yes	No	No	Infected	*Enterobacter cloacae*, gram positive cocci	Rods/Cocci	Ulcer surface
**15**	Yes	Yes	No	Yes	Yes	No	Yes	No	No	Infected	*S. aureus, Pseudomonas aeruginosa*	Rods	Ulcer surface
**16**	Yes	Yes	Yes	Yes	Yes	No	Yes	No	No	Infected	*S.aureus*, gram negative rods	Rods/Cocci	Stratum corneum (cocci), Dermis, Subcutis (rods)
**17**	No	Yes	No	Yes	Yes	No	No	No	Yes	Infected	Gram negative rods	Rods	Dermis, Subcutis
**18**	Yes	Yes	No	Yes	Yes	No	Yes	No	No	Infected	*Pseudomonas aeruginosa*	Rods/Cocci	Stratum corneum
**19**	No	Yes	No	Yes	Yes	No	No	No	Yes	Infected	Mixed growth	Rods/Cocci	Stratum corneum
**20**	Yes	Yes	No	Yes	Yes	Yes	No	No	No	Contaminated	Gram negative rods	None	
**21**	No	Yes	No	Yes	Yes	No	Yes	No	No	Contaminated	*Candida sp*, *Klebsiella pneumonia*	None	
**22**	No	Yes	No	Yes	Yes	No	No	No	No	Contaminated	*Pseudomonas aeruginosa*	n/d	
**23**	No	Yes	No	Yes	No	No	Yes	No	No	Contaminated	Coagulase negative *Staphylococcus species*	n/d	
**24**	Yes	Yes	Yes	Yes	Yes	No	Yes	Yes	Yes	Infected	Acinetobacter sp.	Rods/Cocci	Subcutis
**25**	Yes	Yes	No	Yes	Yes	No	Yes	No	No	Infected	*S. aureus*	Rods	Subcutis
**26**	No	Yes	No	Yes	No	No	No	No	Yes	Infected	*Pseudomonas aeruginosa*	None	
**27**	No	Yes	No	Yes	No	Yes	No	Yes	No	Contaminated	*Providencia stuartii*	None	
**28**	No	Yes	No	Yes	No	No	No	No	No	Contaminated	*Enterobacter cloacae*	n/d	

We compared the clinical presentation to microbiological categorization based on quantification and histological findings. Lesions with a bacterial load less than 10^6^ CFU/ml (or CFU/g) were categorized as contaminated, while lesions with bacterial loads above were considered as infected.

1 WHD = Wound healing delay.

2 SGF = Skin grafting failure.

n/d = not done.

Seven (22.6%) of the 31 lesions clinically suspected to be infected were not confirmed microbiologically by aerobic bacterial count analysis, as the total plate count ranged only between 1.3×10^3^ and 8.9×10^5^ CFU/ml (average 2.7×10^5^ CFU/ml). The remaining twenty-four (77.4%) lesions that were microbiologically confirmed as infected had plate counts ranging between 1.2×10^6^ and 3.5×10^9^ CFU/ml (average value of 1.2×10^9^). *P. aeruginosa* (n = 8; 32%), *P. mirabilis* (n = 5; 20%) and *S. aureus* (n = 3; 12%) dominated among the isolates.

The bacterial load observed in cases analyzed within four weeks post SR8 ranged between 1.3×10^3^ and 4.0×10^9^ CFU/ml; that between five and 12 weeks was between 9.3×10^4^ and 1.2×10^9^ CFU/ml; and that between 9 and 15 months post SR8 ranged between 2.7×10^6^ and 1.8×10^9^ CFU/ml. Nineteen tissue samples and 12 swab samples were analyzed (Table S1) and the bacterial load ranged between 1.3×10^3^ and 4.0×10^9^ CFU/ml for tissues and between 5.2×10^7^ and 2.1×10^9^ for swabs.

**Figure 2:**
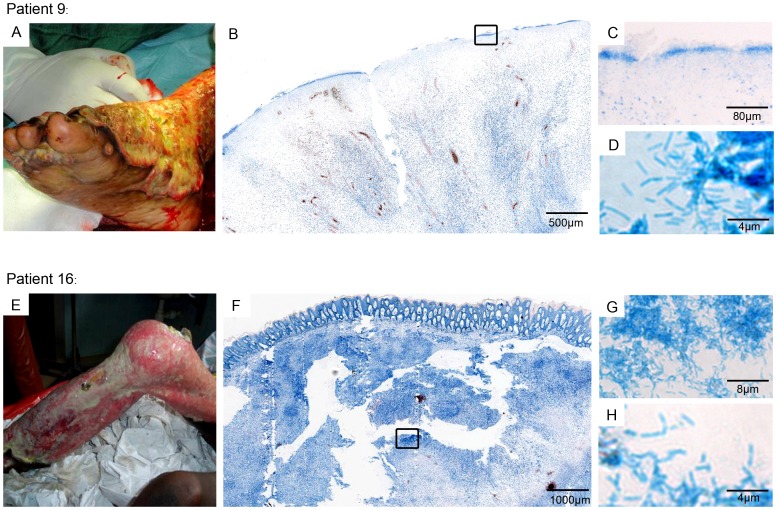
Histopathological analysis of tissue from two patients excised weeks after SR8 treatment respectively. Histological sections were stained with Ziehl-Neelsen (acid fast bacteria) and methylene blue (DNA, secondary infection). A: clinical presentation of patient 9 presenting with a large lesion on the right foot. B: overview over excised tissue specimen (open ulcer surface) revealing the presence of an infection (blue band, box). C/D: higher magnification confirming the presence of densely packed rods. E: clinical presentation of patient 16 presenting with a large lesion covering the left leg. F: overview over excised tissue specimen revealing an epidermal hyperplasia as well as a strong edema. G/H: secondary infection with rods of the dermal and subcutaneous tissue.

Tissue samples from 20/31 of the microbiologically analyzed lesions showing clinical signs of secondary infection after completion of SR8 were also analyzed by histopathology, since the responsible clinicians decided to perform a wound debridement. Microbiological analysis had categorized 16 of these lesions as infected and four as contaminated. None of the microbiologically contaminated wounds presented in the histopathological analysis with a detectable secondary infection. In contrast 12/16 (75%) of the lesions classified microbiologically as infected presented with an infection either with cocci, rods or both (Table 2). Infection was mainly observed in the stratum corneum (6/12; 50%) or on the open ulcer surface (3/12; 25%) and only rarely (3/12; 25%) deeper inside the excised tissue (Table 2). Histopathological analysis of specimen from patient 9 (Figure 2 A–D) revealed a layer of densely packed rods at the open ulcer surface visible already at low magnification as an intensely blue stained band (Figure 2B) At higher magnification, clusters of rod shaped bacteria were observed (Figure 2 C,D). Microbiological analysis confirmed the presence of *P. aeruginosa*. Tissue excised from patient 16 (Figure 2E–H) showed a double infection: cocci being present inside the stratum corneum (data not shown) as well as an extensive infection of the dermal and subcutaneous tissue with rods (Figure F–H). Microbiological analysis isolated *S. aureus* as well as Gram-negative rods. In most of our analysis, histopathological and microbiological results showed a good correlation for most of the patients (Table 2).

### Drug susceptibility pattern of bacterial isolates

Using the disc diffusion assay, a total of 98 Gram-negative rods and Gram-positive cocci obtained from BU wounds were tested for resistance against antibiotics commonly used in Ghana. None of the isolates tested was sensitive to all drugs included in the analysis (Table 3). Five Gram-negative rods were resistant to all tested drugs. More than 70% of the 18 *S. aureus* isolates obtained from infected (n = 12) or contaminated (n = 6) lesions were resistant to flucoxacillin, ampicillin and penicillin. In contrast, 15/18 (83%) were susceptible to gentamicin. The prevalence of *S aureus* isolates resistant to methicillin (MRSA) and vancomycin (VRSA) was 33% and 17%, respectively.

**Table 3:** Antibiotic susceptibility pattern of different bacterial species isolated from BU wounds.

Pathogen	Drug Tested	Number Tested	Susceptible, n(%)	Int. Resistant, n(%)	Resistant n(%)
*Pseudomonas aeruginosa*	Gentamicin	22	18(81.9)	1(4.5)	3(13.6)
	Ceftriaxone	13	3(23.1)	7(53.8)	3(23.1)
	Cefotaxime	20	1(5.0)	1(5.0)	18(90)
	Ampicillin	22	0(0)	0(0)	22(100)
	Tetracycline	22	3(13.6)	1(4.5)	18(81.9)
	Cotrimoxazole	22	3(13.6)	2(0)	17(77.3)
	Cefuroxime	22	0(0)	0(0)	22(100)
	Chloramphenicol	21	2(9.5)	2(9.5)	17(81)
*Staphylococcus aureus*	Tetracycline	18	12(66.7)	0(0)	6(33.3)
	Cotrimoxazole	18	16(88.9)	0(0)	2(11.1)
	Erythromycin	18	9(50)	9(50)	0(0)
	Ampicillin	18	2(11.1)	1(5.6)	15(83.3)
	Flucloxacillin	18	3(16.7)	0(0)	15(83.3)
	Cefuroxime	18	9(50)	1(5.6)	8(44.4)
	Gentamicin	18	15(83.3)	0(0)	3(16.7)
	Methicillin	18	12(66.7)	0(0)	6(33.3)
	Vancomycin	18	15(83.3)	0(0)	3(16.7)
	Penicillin	18	0(0)	0(0)	18(100)
Other gram positive	Tetracycline	13	6(46.2)	0(0)	7(53.8)
	Cotrimoxazole	13	7(53.8)	0(0)	6(46.2)
	Erythromycin	13	6(46.2)	3(23.1)	4(30.7)
	Ampicillin	13	3(23.1)	1(7.7)	9(69.2)
	Flucloxacillin	13	2(15.4)	0(0)	11(84.6)
	Cefuroxime	13	6(46.2)	0(0)	7(53.8)
	Gentamicin	13	11(84.6)	0(0)	2(15.3)
	Penicillin	13	2(15.4)	0(0)	11(84.6)
Other gram negatives	Gentamicin	45	37(82.2)	1(2.2)	7(15.6)
	Ceftriaxone	17	10(58.8)	2(11.8)	5(29.4)
	Cefotaxime	39	16(41.0)	4(10.3)	19(48.7)
	Ampicillin	45	0(0)	1(2.2)	44(97.8)
	Tetracycline	45	1(2.3)	0(0)	44(97.8)
	Cotrimoxazole	45	7(15.6)	0(0)	38(84.4)
	Cefuroxime	45	5(11.1)	10(22.2)	30(66.7)
	Chloramphenicol	39	6(15.4)	2(5.1)	31(79.5)

Likewise most of the *P. aeruginosa* strains were resistant to most of the tested drugs. However, most isolates (18/22; 82%) were susceptible to gentamicin. Results for the other Gram-negative and -positive bacteria are provided in Table 3.

### Microbiological analysis of recycled bandages

When monitoring wound management procedures, it was realized that patients and care-givers were instructed by health workers to wash and recycle dressing bandages. We therefore conveniently sampled dressings that have been used and washed for the next dressing. Seventeen bandages from fifteen BU cases were analyzed and as shown in Table 4, all of them had some bacterial contamination with total aerobic plate counts ranging between 2.2×10^3^ and 3.2×10^8^ CFU/g with an average count of 2.8×10^7^ and a median value of 1.2×10^5^ CFU/g. While bacterial species identified included commensals such as staphylase negative *Staphylococcus spp.*, also potential pathogens including *S. aureus*, *P. aeruginosa*, *Flavibacterium oryzihabitans*, *Enterobacter agglomerans* and *Enterobacter cloacae* were isolated. The drug susceptibility patterns of isolates are indicated in Table 4. Similar isolates were also isolated from patients' wounds.

**Table 4:** Microbiological analysis of recycled bandages.

CASE	BACTERIAL LOAD (CFU/g)	ORGANISM ISOLATED	ANTIBIOTIC SUSCEPTIBILITY		
			SENSITIVE	INTERMEDIATE	RESISTANT
CASE 1	9.5×10^7^	*Enterobacter agglomerans*	CTX, TET, AMK, COT, GEN, CHL		AMP, CRX
CASE 2	5.3×10^7^	*Staphylococcus warneri*	COT, CRX, GEN	ERY	PEN, AMP, FLX, TET
CASE 3	5.5×10^4^	*Staphylase negative Staphylococcus*	TET, COT, CRX, GEN	ERY	PEN, AMP, FLX
CASE 4	1.10×10^6^	*N/D*			
CASE 5	3.2×10^8^	*Staphylase positive Staphylococcus*	TET, COT, CRX, GEN		PEN, AMP, FLX, TET
CASE 6	1.22×10^5^	*N/D*			
CASE 7	1.67×10^6^	*Flavibacterium oryzihabitans*	TET, AMK, GEN	CRX, CTX	AMP, COT, CHL
CASE 8	8.6×10^5^	*Staphylase negative Staphylococcus*	GEN		PEN, AMP, FLX, ERY, TET, COT, CRX
		*Pseudomonas sp*	CTX, TET, COT, CHL		AMP, CRX, AMK,GEN
CASE 9	4.1×10^3^	*Staphylase negative Staphylococcus*	TET, COT, CRX, GEN		PEN, AMP, FLX, ERY
CASE 10	3.3×10^5^	*Staphylase negative Staphylococcus*	GEN	CRX	PEN, AMP, FLX, ERY, TET, COT
CASE 11	3.1×10^3^	*Staphylase negative Staphylococcus*	TET, GEN	CRX	PEN, AMP, FLX, ERY, COT
CASE 12	6.3×10^4^	*Staphylase negative Staphylococcus*	TET, COT, CRX, GEN	ERY	PEN, AMP, FLX
CASE 13	1.65×10^5^	*Enterobacter cloacae*	CTX, TET, COT, GEN, CHL	AMK	AMP, CRX
CASE 14A	4.4×10^4^	*Staphylase positive Staphylococcus*	TET, GEN	CRX	PEN, AMP, FLX, ERY, COT
CASE 14B	5.3×10^3^	*Staphylase negative Staphylococcus*	GEN		PEN, AMP, FLX, ERY, TET, COT, CRX
CASE 15A	NEGLIGIBLE	*Staphylase negative Staphylococcus*	GEN	ERY, CRX	PEN, AMP, FLX, TET, COT
CASE 15B	NEGLIGIBLE	*N/D*			

*AMP = Ampicillin, CXM = Cefixime, CXC = Cloxacillin, COT = Cotrimoxazole, ERY = Erythromycin, GEN = Gentamicin, TET = Tetracycline, PEN = Penicillin, CRX = Cefuroxime, CHL = Chloramphenicol, CTR = Ceftriaxone, CTX = Cefotaxime*.

## Discussion

Mycolactone, the cytotoxic macrolide toxin of *M. ulcerans* plays a key role in the pathology of BU. It causes apoptosis of mammalian cells [8], [27] and has immunomodulatory activity [28], [29]. Since a number of macrolides have antibiotic activity against a broad spectrum of bacteria, including streptococci, pneumococci, staphylococci, enterococci, mycoplasma, mycobacteria, rickettsia, and chlamydia [30], it has been speculated that mycolactone secreted by *M. ulcerans* during active disease prevents secondary bacterial infections of BU lesions. The goal of this study was to find out whether ulcerative BU lesions are indeed rarely colonized or infected by other bacterial species. To address this, BU wounds were characterized before SR8 treatment by both direct smear microscopic analysis for the presence of bacteria and neutrophils [20] and by pour plate determination of aerobic CFU counts. More than 60% of the lesions tested before treatment had bacterial counts ≥10^6^ CFU/ml and direct smear examination frequently showed large numbers of bacteria and neutrophils (Figure S1). A broad spectrum of bacterial species was isolated from the lesions with *S. aureus*, *P. aeruginosa* and *P. mirabilis* being the most frequently found species. This suggests that *M. ulcerans* infection and mycolactone secretion does not prevent secondary bacterial infections.

Chronic wounds often have a bacterial burden that is massively exceeding levels used to define lower limits for the definition of infection in acute surgical wounds (i. e. 10^6^ CFU/g of tissue). However, many chronic wounds go on to closure despite levels of infecting microorganisms ≥10^8^ CFU/g of tissue, with infection by Group B streptococci being one exception to this rule [12], [13], [16]. Because of the intrinsic differences in the way acute and chronic wounds respond to the burden of microorganism, emphasis is currently being placed on holistic assessments, with clinical signs and symptoms playing key roles in the diagnosis of chronic wound infection. Clinical signs usually employed for diagnosis include erythema, edema, heat, purulent exudates with concurrent inflammation, pain, delayed healing, discoloration of granulation tissue, friable granulation tissue, pocketing at the base of the wound, foul odor, and wound breakdown [13], [14], [17]. In particular increasing pain and wound breakdown have been shown to be good predictors of infection in chronic wounds. In this study we combined clinical, histopathological, qualitative and quantitative microbiological methods to analyze BU lesions for the presence of infections after completion of SR8 treatment. Lesions from 28 patients showing clinical signs of infection were included in this analysis. 75% of these lesions yielded CFU counts >10^6^ CFU/ml (average value of 1.2×10^9^) and frequently species with pathogenic potential, such as *S. aureus*, *P. aeruginosa*, *S. haemolyticus*, *E. cloacae* and *K. pneumonia* were isolated. Pain and yellow discharge turned out to be highly predictive clinical indicators for infection. For the patients that had clinical signs of infection after SR8, culture and drug susceptibility testing results were submitted to the treating officer. However documentation of the treatment and subsequent follow-up of patients was beyond the scope of this study.

A study analyzing the microbial flora of healing and non-healing decubitus ulcers [31] found *S. aureus*, *Streptococcus spp.*, *E. coli*, *Klebsiella spp.*, *Proteus spp.* and *P. aeruginosa* as the main organisms that caused infection of the ulcers. Chronic venous ulcers have been found to be infected with *S. aureus*, *P. aeruginosa*, Coagulase-negative staphylococci, *Proteus spp.* and anaerobic bacteria [32]. Thus most of the organisms isolated in this study from BU lesions have also been found associated with infection of other types of wounds. Similar to what has been reported in other studies [33], lesions were in many cases infected with more than one bacterial species (Table 2). Our data on the microflora of lesions upon admission indicate that BU lesions may be contaminated from the communities as a result of improper wound care practices by the patients in their quest to treat the infection either on their own or with the help of traditional healers or herbalists. There is major concern about subsequent acquisition of antibiotic resistant organisms from the hospital settings. After the present pilot study demonstrating colonization and infection during and after SR8 treatment, we plan to perform longitudinal studies with patient cohorts to study the influence of BU wound management practices on secondary bacterial infections.

The method used for collecting wound specimens can influence the data obtained from microbiological culturing. Currently, collection of a biopsy specimen is the gold standard for determining the presence and identity of microorganisms within the wound bed tissue [12], [16], [34]–[37]. However, there are limitations as to which healthcare providers can collect biopsies, the availability of laboratories offering microbiological culture testing on biopsies, the expenses involved with the performance of these tests, and the potential for further tissue damage and delay of wound healing when biopsies are taken. In the present study we employed swabbing [22], [36] as the main sampling procedure and performed histopathological studies with tissue specimen only from 20 cases that underwent surgical intervention. The histopathological analysis detected bacterial populations in 75% (12/16) of the analyzed lesions classified as infected and in none (0/4) of the lesions classified as contaminated. This strong correlation between results obtained with tissue and swab samples confirms results of previous studies [23] indicating that microbiological swabbing is a good sampling procedure for the determination of infection of wounds. Histopathological analysis detected infecting bacteria populations only rarely deeper inside the excised tissue and mainly in the stratum corneum or on the open ulcer surface, where bacteria are accessible for the swabs.

Contamination of BU lesions prior to SR8 treatment may be a result of wound care practices by the patients. Also during SR8 treatment a range of bacterial species, with Gram-negative rods dominating, were isolated from the lesions. This indicates that SR8 does not necessarily eliminate contamination or secondary infection of lesions. Bacterial species, such as *P. aeruginosa, K. pneumoniae and S. aureus* isolated from infected lesions after completion of SR8 treatment, may however also have been acquired from the hospital setting. A detailed characterization of isolates is required to address this important issue further. Both mono and multiple antibiotic resistant strains were isolated with high frequency from the BU lesions. For example all the tested *S. aureus* strains were resistant to penicillin, 22% were methicillin resistant and 17% vancomycin resistant. Dependent on the setting, both lower (Nigeria, [38]) or higher (South-Africa, [39]) frequencies have been reported in Africa. Most worrying in this context is the high (83%) level of resistance of *S. aureus* isolates to flucloxacillin, which is in Ghana the main antibiotic in use for treating skin infections such as boils and cellulitis. In addition, we acknowledge that true VRSA is rare, and that the occurrence of apparent VRSA is being followed up through referral of isolates to an international reference laboratory.

Postoperative infections of wounds represent the commonest surgical complication causing substantial increases in the duration and costs of hospital stays [40]. Our pilot study involving BU patients at different time points of SR8 treatment indicates that secondary bacterial infection may be a prominent cause for delays in wound healing and skin grafting failures. These findings call for an optimization of BU wound management and hygiene procedures to better control secondary infections. Also the choice of treatment of secondary infections with locally available antimicrobial agents requires a better understanding of the infecting flora and of drug susceptibility patterns. Our study did not follow the same patients from beginning of treatment till they were healed and this has limited the ability to determine causes and consequences of wound infection. More studies are required to ascertain the impact and source of wound infection in SR8 treatment of BU and to support development of guidelines for wound care in BU case management. In addition to wounds we also analyzed bandages that have been washed by the patients themselves to be re-used for wound dressing. From these bandages we isolated potential wound pathogens including *S. aureus*, *P. aeruginosa*, *Flavibacterium oryzihabitans*, *Enterobacter agglomerans* and *Enterobacter cloaca*; thus the bacteria profile of the wound samples was comparable to that of the bandages. These findings indicate that the recycling of bandages may not be a good practice as it may be one of the sources of wound infection. We recommend that if for economical reasons bandages need to be recycled, they must be washed well with an appropriate disinfectant.

## Supporting Information

Figure S1 **Direct smear examination of infected wounds and Kirby-Bauer plate of a VRSA isolate.** Exudates from infected wounds were smeared directly over clean microscopic slides. The slides were then stained by the Gram procedure and viewed under oil immersion. While the exudate on Plate A is derived from the lesion of the patient whose biopsy was analyzed by histopathology before SR8 (Figure 1), the smears on plate B and C were taken from cases after SR8 treatment. Plate D depicts the drug susceptibility result of two *S. aureus* isolates. While one strain is both methicillin and vancomycin resistant, the other is methicillin resistant, but vancomycin susceptible.
(TIF)

Table S1 **Samples taken and types of analysis conducted at various stages of treatment.**
(DOC)
