# Supplementary figures and images for: Secondary Bacterial Infections of Buruli Ulcer Lesions Before and After Chemotherapy with Streptomycin and Rifampicin
Source: PLoS Negl Trop Dis. 2013 May 2;7(5):e2191. doi: 10.1371/journal.pntd.0002191 (PMC3642065; doi:10.1371/journal.pntd.0002191)

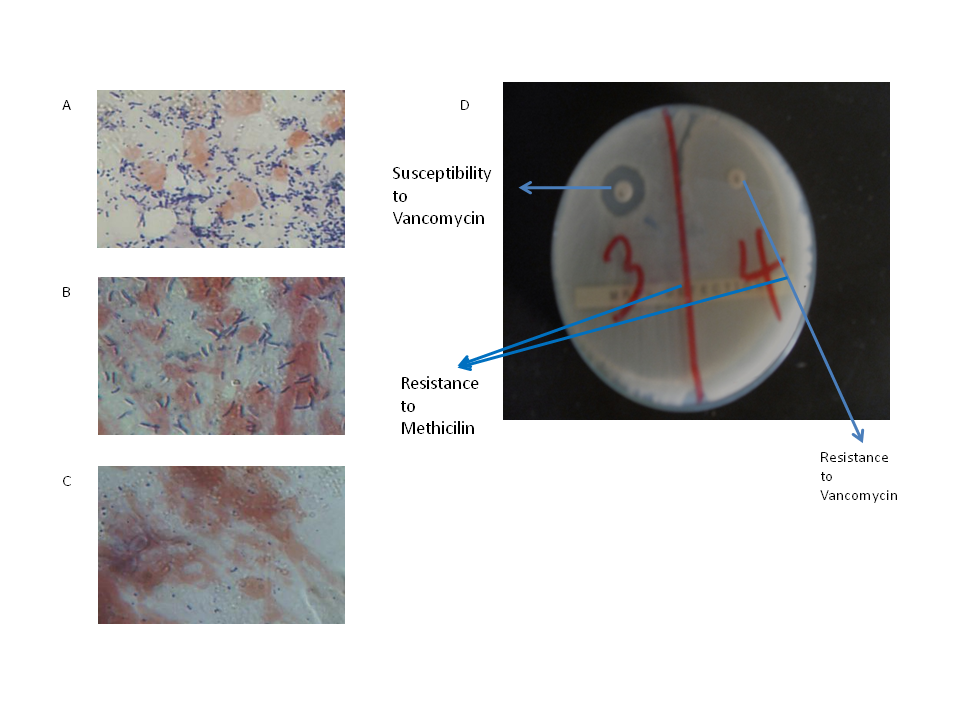

Supplement: Figure S1 — Direct smear examination of infected wounds and Kirby-Bauer plate of a VRSA isolate. Exudates from infected wounds were smeared directly over clean microscopic slides. The slides were then stained by the Gram procedure and viewed under oil immersion. While the exudate on Plate A is derived from the lesion of the patient whose biopsy was analyzed by histopathology before SR8 (Figure 1), the smears on plate B and C were taken from cases after SR8 treatment. Plate D depicts the drug susceptibility result of two S. aureus isolates. While one strain is both methicillin and vancomycin resistant, the other is methicillin resistant, but vancomycin susceptible. (TIF) [file pntd.0002191.s001.tif]
