# Supplementary material for: Secondary Bacterial Infections of Buruli Ulcer Lesions Before and After Chemotherapy with Streptomycin and Rifampicin
Source: PLoS Negl Trop Dis. 2013 May 2;7(5):e2191. doi: 10.1371/journal.pntd.0002191 (PMC3642065; doi:10.1371/journal.pntd.0002191)
Supplement: Table S1 — Samples taken and types of analysis conducted at various stages of treatment. (DOC) [file pntd.0002191.s002.doc]

| **Types of samples and analysis performed** | **Before treatment** | **During treatment** | **After treatment** |
| --- | --- | --- | --- |
| Swab samples used for microbiology | 52 | 20 | 12 |
| Biopsy samples used both for histopathology and microbiology | 1 | 0 | 19 |
| Biopsy samples used only for histopathology | 0 | 0 | 1 |
